# Different nucleosomal architectures at early and late replicating origins in *Saccharomyces cerevisiae*

**DOI:** 10.1186/1471-2164-15-791

**Published:** 2014-09-13

**Authors:** Ignacio Soriano, Esther C Morafraile, Enrique Vázquez, Francisco Antequera, Mónica Segurado

**Affiliations:** Instituto de Biología Funcional y Genómica, Consejo Superior de Investigaciones Científicas, Universidad de Salamanca (CSIC/USAL), Campus Miguel de Unamuno, Salamanca, 37007 Spain; Departamento de Microbiología y Genética, (USAL), Campus Miguel de Unamuno, 37007 Salamanca, Spain

## Abstract

**Background:**

Eukaryotic genomes are replicated during S phase according to a temporal program. Several determinants control the timing of origin firing, including the chromatin environment and epigenetic modifications. However, how chromatin structure influences the timing of the activation of specific origins is still poorly understood.

**Results:**

By performing high-resolution analysis of genome-wide nucleosome positioning we have identified different chromatin architectures at early and late replication origins. These different patterns are already established in G1 and are tightly correlated with the organization of adjacent transcription units. Moreover, specific early and late nucleosomal patterns are fixed robustly, even in *rpd3* mutants in which histone acetylation and origin timing have been significantly altered. Nevertheless, higher histone acetylation levels correlate with the local modulation of chromatin structure, leading to increased origin accessibility. In addition, we conducted parallel analyses of replication and nucleosome dynamics that revealed that chromatin structure at origins is modulated during origin activation.

**Conclusions:**

Our results show that early and late replication origins present distinctive nucleosomal configurations, which are preferentially associated to different genomic regions. Our data also reveal that origin structure is dynamic and can be locally modulated by histone deacetylation, as well as by origin activation. These data offer novel insight into the contribution of chromatin structure to origin selection and firing in budding yeast.

**Electronic supplementary material:**

The online version of this article (doi:10.1186/1471-2164-15-791) contains supplementary material, which is available to authorized users.

## Background

Eukaryotic chromosomes are replicated from multiple replication origins. In budding yeast, replication origins are termed ARS (autonomously replicating sequences) and contain the ARS consensus sequence (ACS), which is essential for origin activity [1]. During the G1 phase, pre-replicative complexes (pre-RCs), composed of the origin recognition complex (ORC), Cdc6, Cdt1, and an inactive form of the replicative helicase Mcm2-7 complex are assembled into ACS-containing origin regions, a process called ‘licensing’ [2]. During S phase, origin activation is induced by the activation of the MCM2-7 helicase and by the recruitment of further initiation factors, including Mcm10, Cdc45, GINS, Sld2, Sld3 and Dpb11, to form pre-initiation complexes (pre-IC) [3]. Although all replication origins are ‘licensed’ during G1 phase [4, 5], not all of them are activated during the subsequent replication cycle and, moreover, origins are activated at different times along S phase. The temporal order of origin activation is called the replication timing program, and it is established in G1 phase [6].

The mechanisms orchestrating the timing program are not fully understood, but many studies have indicated that the firing of origins can be influenced by different factors involving replication initiation mechanisms, epigenetic modifications, chromosomal position and the chromatin context [7–17].

Limiting levels of initiation factors have been reported [15, 16], and constitute a point of control of replication initiation. The amount of Sld3, Sld2, Sld7, Dpb11, Cdc45 and Dbf4 factors is below the number of “licensed” origins, and hence activation is necessarily restricted to a subset of origins. Some of these factors, i.e. Sld3 and Cdc45, can be detected specifically at some early firing origins during G1 [11, 18, 19], which is critical for promoting their activation at the beginning of S phase. However, even though the sequential activation of origins can be explained by limiting levels of essential initiation proteins, an open question is why limiting factors are preferentially recruited by early origins.

Recent reports have indicated that the Forkhead transcription factors Fkh1 and Fkh2 are involved in early origin firing [11, 17]. Although *FKH1* and *FKH2* are not required for origin licensing, the binding of Cdc45 to early origins in G1 seems to be Fkh1/2-dependent, and it has been suggested that the role of Fkh1/2 in origin firing would involve the establishment of replication timing domains and the spatial organization of origins [11]. Fkh1/2 binding sites are enriched in a subset of origins, but ChIP–chip analyses indicate that there is no strict correlation between the presence of consensus motifs and Fkh1/2 binding [20, 21]. In addition, the presence of Fkh1/2 binding sites is not sufficient to confer early firing, and the number and distribution of Fkh motifs within the origin region seem to be important for conferring early origin activation [11, 17], arguing for an additional role of the chromatin environment in the regulation of origin firing.

Chromatin modifications also play a role in the regulation of origin timing and several studies have demonstrated that increases in histone acetylation, either by deletion of the deacetylase *RPD3* or by artificial recruitment of the histone acetylase Gcn5, correlate with advanced origin firing [9, 10, 12, 22]. However, the explanation as to how histone acetylation modifies replication timing has remained obscure.

Previous studies have shown that replication origins in budding yeast exhibit a specific nucleosomal configuration, consisting of a nucleosome-depleted region (NDR) next to the ACS element, flanked by well-positioned nucleosomes [23, 24]. The correct organization of these origin-associated chromatin features is important for efficient replication initiation [25] and it would also be important for the timing of origin activation.

In light of the above, here we examined the contribution of the chromatin structure to the timing of origin firing by genome-wide analysis of the nucleosome organization at early and late origins and its relationship with epigenetic modifications, in particular with histone acetylation. Our findings show that early and late origins exhibit different nucleosomal patterns, which are influenced by the genomic context. We found that higher histone acetylation in *rpd3* mutants was linked to local changes in chromatin structure, leading to a more open conformation at the affected replication origins. Finally, our analysis indicates that origin structure is dynamic during the cell cycle.

## Results

### Early and late replication origins exhibit different nucleosome profiles

**Figure 1:**
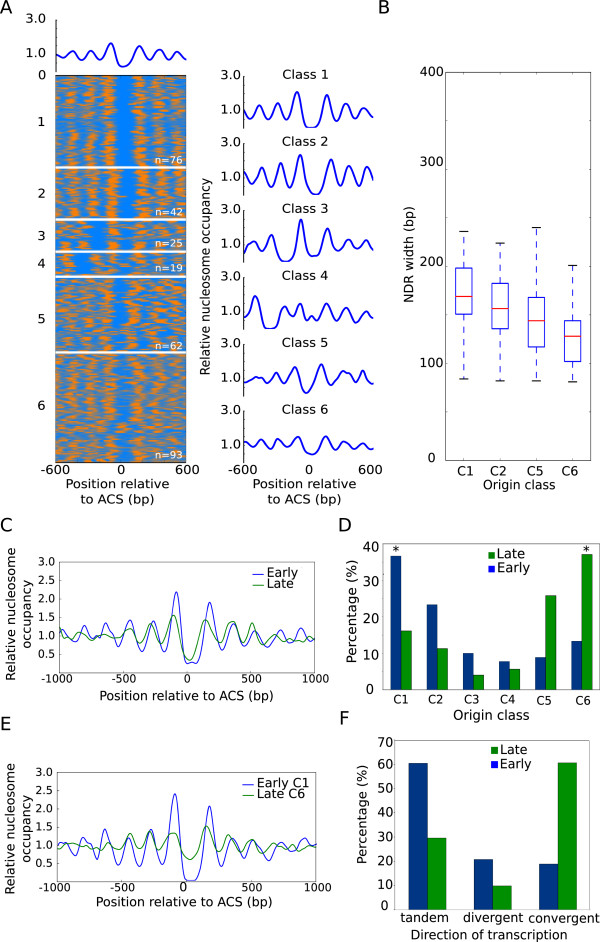
**Early and late replication origins show different nucleosomal organization patterns. (A)**, Heatmap of nucleosome occupancy from 317 replication origins. ARSs are aligned on the Y axis, and the distance from the ACS is indicated on the X axis. Nucleosome occupancy is indicated in orange and nucleosome depletion is indicated in blue. Average nucleosome profiles for the six groups from the k-means clustering are plotted on the right (blue line). **(B)**, Box plot analysis of the NDR width for origin classes shown in **A**. Classes 3 and 4 were excluded due to their reduced sample size. The bottom and top of the box are the first and third quartiles, and the red band inside the box is the second quartile (the median). The ends of the whiskers represent the lowest and highest data still within 1.5 IQR of the lower and upper quartiles respectively. **(C)**, Comparative nucleosomal profiles of early (blue line) and late (green line) origin groups. **(D)**, Percentage of early (blue) and late (green) replication origins included in the classes established in **A**. **(E)**, Comparative nucleosomal profiles of class 1-early origins (blue line) and class 6-late origins (green line). **(F)**, Percentage of early (blue) and late (green) replication origins located in intergenic regions between tandem, divergent and convergent transcripts.

To study the interplay between chromatin organization and replication timing, we generated high-resolution maps of nucleosome positions of the entire genome of *S. cerevisae* (see Methods). Previous experiments indicate that origin timing is established in early G1 phase [6] and we therefore determined the nucleosomal profile of replication origins from G1-synchronized cells (Figure 1A). Given the importance of aligning nucleosome maps relative to ACS to uncover chromatin structure in origins [23, 24], we selected a subset of 317 origins with previously annotated ACS [26] (Additional file 1: Table S2). As shown in Figure 1A (top panel), the average nucleosome profile of replication origins is characterized by the presence of an NDR that covers the ACS element asymmetrically, flanked by well-positioned nucleosomes at both sides, as previously reported [23, 24]. However, a diversity of chromatin structures in replication origins has been reported [23], and to identify variations in their nucleosomal patterns we performed a k-means clustering analysis to classify origins by their similarities in the NDR width and degree of occupancy of the flanking nucleosomes. Following those criteria, six origin groups with relatively distinct patterns of nucleosome distribution were chosen (see Methods) (Figure 1A, heat map). From classes 1 to 6, the NDR width diminished from 193 to 129 bp as did the positioning and occupancy of the nucleosomes located at adjacent positions (Figures 1A and 1B).

Since replication origins displayed different types of nucleosomal configuration as well as a different timing of activation, we wondered whether early and late origins might be preferentially linked to some of the nucleosome profiles identified above. First, we independently aligned nucleosome maps corresponding to early and late origins groups relative to the ACS. The assignment of replication origins within early or late categories for our analysis was made according to timing data available from at least three independent studies (OriDB; [12, 14, 27–29] (Additional file 1: Table S2). Comparison of their average nucleosome profiles showed that early origins displayed a higher occupancy of nucleosomes immediately upstream (-1) and downstream (+1) from a broader NDR feature, along with a better positioning of the adjacent nucleosomes (Figure 1C).

Second, we examined the nucleosomal arrangement of early and late replication origins within the six types of nucleosome patterns described above. Although the 6 different classes of origin organization were found within early and late origin groups (Additional file 2: Figure S1A), quantification of the distribution of early and late origins between the different classes revealed a marked preferential distribution of early origins for classes 1 and 2 and of late origins for classes 5 and 6 (Figure 1D). Indeed, 60% of early origins belonged to classes 1 and 2, while 65% of late origins belonged to classes 5 and 6. Moreover, nearly 40% of early or late origins were clustered in class 1 or 6 respectively (asterisks in Figure 1D). The overlap between the profiles of classes 1 and 6, which include the highest proportion of early and late origins, shows a striking difference in chromatin architecture between them (Figure 1E).

Next, we wondered whether early and late origins, or their different chromatin structure, could be influenced by the genomic context in which origins are placed. In *S. cerevisiae*, replication origins are mainly located at intergenic regions (IGR), which may be flanked by genes transcribed co-directionally, convergently or divergently. Replication origins in budding yeast appeared in these three types of IGR, but when we quantified the number of early and late origins located in each type of IGR, we found that they were not randomly distributed between them. In fact, while early origins can be found in all types of IGR with nearly the expected probability, late origins showed a strong bias for convergent IGR (Additional file 2: Figure S1B).

We asked whether this preferential localization would be related to their different chromatin architecture. To address this question we quantified the number of C1&2-early and C5&6-late origins located in each type of IGR, and we found that 60% of origins in each group mapped to IGR between tandem and convergent transcripts, respectively (Figure 1F). Moreover, class 6 was particularly enriched in convergent IGR, and the percentage of C6-late origins at this type of IGRs reached 71%. Our results indicated that class 6 was linked to this type of gene organization (convergent transcription), and class 6 is highly enriched with late origins, therefore late origins are necessarily over-represented at convergent IGR. Origin-free NDR in convergent IGR were on average shorter and presented lower nucleosome occupancy than NDR located in IGR with gene units organized in tandem or divergently (Additional file 2: Figure S1C), resembling class 6-origin patterns. This might suggest that IGR with convergent transcription are prone to have lower nucleosome occupancy and shorter NDR.

In sum, although a nucleosome-depleted region next to the ACS element flanked by positioned nucleosomes is a common feature shared by all origins, the above analysis identified previously unknown separate modes of nucleosome organization in early and late replication origins. In addition, these early and late-nucleosomal patterns were preferentially located in different kinds of intergenic regions, which might condition their different chromatin structure and timing.

### Fkh-binding sites co-localize with the NDR in early origins

**Figure 2:**
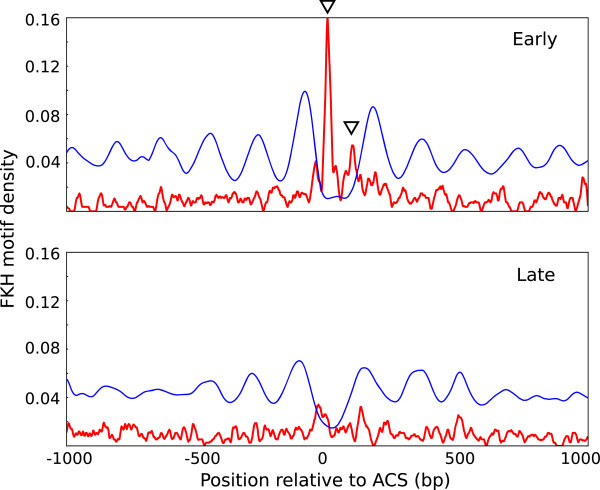
**Fkh-binding sites are enriched in the NDR of early origins.** Aggregated nucleosomal profiles (blue line) and Fkh-motif density (red line) were aligned relative to the ACS of early (top panel) and late (bottom panel) replication origins.

Next, we investigated the relationship between factors involved in replication timing and chromatin organization. A role of Fkh1/2 transcription factors in early origin firing has been described [11, 17]. However, although Fkh1/2-binding sites are widespread throughout the genome and are frequently found in origins, only Fkh1/2 consensus sites placed in very close proximity to ACSs confer early origin activation [11, 17]. In fact, it has been recently reported that some early origins present two Fkh1/2-binding sites separated not less than 60 bp and not more than 120 bp [17], although the reason behind that is unknown. We argued that the distribution of Fkh1/2 motifs might be related to origin architecture.To examine this possibility, we inspected the location of Fkh1/2-binding sites in replication origins in the context of chromatin organization. Thus, we searched for the Fkh-binding sequence throughout the genome and plotted the average enrichment of the Fkh motif in early or late origin clusters (red line) within the framework of their nucleosome profiles (blue line). Figure 2 shows that the Fkh-binding sequence was highly represented only in early origins, as expected. In addition, our analysis indicated that there were two preferential Fkh-binding sites at early origins, and remarkably, both sites showed a precise co-localization with the NDR. These two Forkhead sites are located at each border of the NDR; the best represented is located in close proximity to ACS, while the other one is on average 80–100 bp apart, beyond a middle NDR region where Fkh sites are excluded.

These results advance our previous knowledge by showing that Fhk1/2 motifs are often positioned at the boundaries of the NDR, which could explain why critical Fkh1/2 motifs are restricted to the close vicinity of ACS and why they are not further apart than 120 bp. Therefore, the localization and distribution of Fkh1/2 motifs required for early origin function might be imposed or determined by the NDR element.

These data also suggest that the location of Fkh motifs within accessible chromatin is important for early origin firing. It would be possible that the higher accessibility of Fkh1/2 motifs located within the NDR may favour the binding of Fkh1/2 factors, or/and the interaction between Fkh and replication factors such as Cdc45 in early origins [11, 17]. However, not all early origins contain Fkh-binding sites, and only 25.6% of the early origins considered in our analysis had this specific configuration of 2 Fkh sites close to the ACS, indicating that other factors must influence early firing too.

### An increase in histone acetylation correlates with local nucleosomal remodelling in *rpd3*mutants

Histone modifications also play a role in determining origin timing, and an increase in histone acetylation around origins induces earlier firing [9]. Our data revealed a correlation between origin structure and replication timing and we therefore wondered whether histone acetylation might modify replication timing by altering the chromatin architecture of replication origins. To examine this possibility, we generated genome-wide nucleosome maps in an *rpd3Δ* mutant, where the *RPD3* histone deacetylase gen had been deleted.

**Figure 3:**
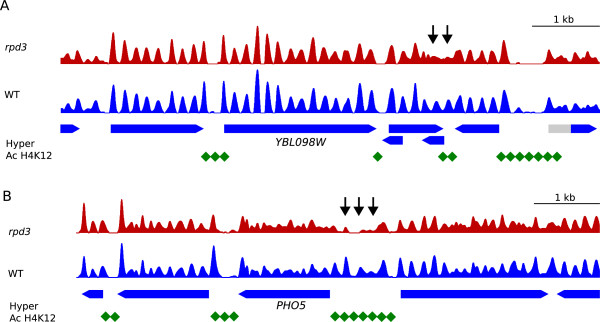
**Histone deacetylation in the absence of Rpd3 correlates with local modifications of nucleosome organization. (A)**, Nucleosome patterns across a genomic region in chromosome II from WT (blue) and *rpd3Δ* cells (red). Blue pointed rectangles represent genes where the coding (blue) and non-coding (grey) regions are indicated. Black arrows indicate nucleosome remodelling in the absence of Rpd3. Green diamonds represent the hyperacetilated regions in the mutant cells (Robyr et al., [31]). **(B)**, As in B but for the *PHO5* gene region.

Despite Rpd3 playing a key role in the global deacetylation of the two core histones H3 and H4 [30], our maps showed that the general pattern of nucleosome distribution was mostly unaltered in the absence of Rpd3 (Figure 3 and Additional file 3: Figure S2). In fact, the average nucleosome organization of genes relative to the +1 nucleosome or relative to the Transcription Start Site (TSS) was very similar in the wild-type and *rpd3Δ* mutant strains (Additional file 3: Figure S2A). However, the analysis of individual genomic regions showed that although most nucleosomes occupied the same positions in both strains, some differences were observed at specific nucleosomes (arrows in Figure 3A and Additional file 3: Figure S2B), as in the case of the acid phosphatase *PHO5* locus. Deletion of *RPD3* increases the expression of the *PHO5* gene and involves deacetylation along a 4.25-kb region that includes *PHO5* [30]. However, chromatin remodelling in the absence of Rpd3 was restricted to a few nucleosomes surrounding an NDR located upstream from the 5′ end of the gene (Figure 3B). This observation indicated that histone deacetylation results in local modifications of nucleosome organization in key regulatory elements, which seem to have a great impact on transcriptional regulation, and perhaps also on replication timing.

### Deletion of the deacetylase *RPD3*increases origin accessibility

To explore the above possibility, we wondered whether chromatin structure in origin regions would also be modified locally in an Rpd3-dependent manner. Analysis of the average nucleosomal organization of replication origins from G1-synchronized cells showed that origins presented a NDR flanked by well-positioned nucleosomes in *rpd3Δ* cells too and, also, that early and late origins displayed different nucleosome profiles when analyzed separately (Additional file 4: Figure S3A). This result indicated that the different profiles observed at early and late origins were Rpd3-independent.

**Figure 4:**
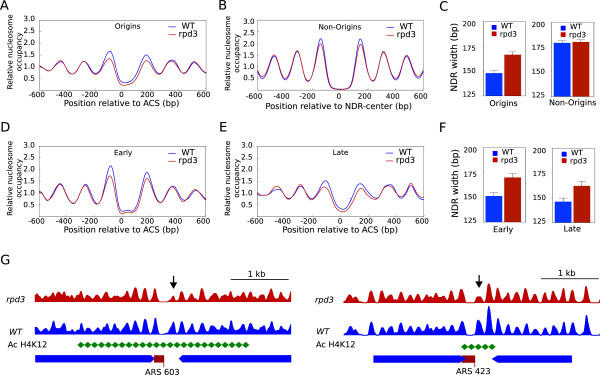
**Origin accessibility is increased in the absence of Rpd3.** Aggregated nucleosomal profiles of 318 NDR associated with replication origins **(A)** or 320 NDR non-associated with origins **(B)** from G1-arrested WT (blue) and *rpd3Δ* (red) cells were aligned relative to the ACS or to the NDR center, respectively. **(C)**, Average NDR width of replication origins (left graph) or non-origin regions (right graph) from WT (blue) and *rpd3Δ* mutants (red). **(D)**, As in A but for early origins. **(E)**, As in A but for late origins. **(F)**, As in C but for early (left graph) and late origins (right graph). **(G)**, Nucleosome patterns around the late origins ARS 603 and ARS 423 from WT (blue) and *rpd3Δ* samples (red). Hyper-acetylated regions in the *rpd3* mutant (Robyr et al., [31]; Vogelauer et al. [9]) are represented in green. Black arrows point to nucleosomes affected in the absence of Rpd3.

However, when we compared the nucleosome profile of replication origins between the wild-type and *rpd3Δ* strains, we observed that the chromatin structure of origins was affected in the absence of Rpd3 in G1 (Figure 4A), but not in S-phase (Additional file 4: Figure S3B). Thus, *rpd3* mutants had a wider NDR and lower occupancy of the -1 and +1 nucleosomes. Importantly, the average nucleosome pattern in NDR that did not colocalize with origins revealed no significant changes between the two strains (Figure 4B), indicating that the observed Rpd3-dependent remodelling was origin-specific. To estimate the extent of the differences in the NDR length between wild-type and *rpd3Δ* strains, we measured the width of the NDR of replication origins individually and plotted the average length for each strain. Figure 4C shows that the NDR size increased in *rpd3Δ* mutants in origins, but not in the control group (see also Additional file 1: Table S2).

To discriminate whether the G1-specific nucleosomal remodelling observed was affecting early or late replication origins or both, we analyzed the average nucleosome profiles of early and late origin groups independently. Figure 4D-E shows that chromatin structure was remodelled in both types of origins, although changes in the NDR width and occupancy in late origins were slightly more pronounced. According to this, the NDR size increased in *rpd3Δ* mutants in both early and late origins (Figure 4F). Local modifications in the NDR region also included the aperture of an NDR that was occupied by a nucleosome in a wild-type strain. This NDR “opening” was taken place in the absence of Rpd3 in 21% of early and 39.2% of late origins with these characteristics, and an example is shown in Additional file 4: Figure S3D for ARS1410.

Chromatin remodelling in the de-acetylation mutant also affected the occupancy of nucleosomes flanking the NDR. These local modifications were examined in individual origin regions in the absence of Rpd3, and some origins such as ARS603 and ARS423 showed a noticeable decrease in occupancy in the +1 nucleosome (arrows in Figure 4G) while in others like ARS1413 the nucleosomes -1, -2 and -3 were also affected (Additional file 4: Figure S3E). When acetylation data were available [9, 31] for the origin analyzed, we found that the chromatin-remodelled region was hyper-acetylated (Figure 4G).

Our data indicate that Rpd3 affects the nucleosomal configuration of many early and late origins. Similarly, Rpd3 affects the replication timing of numerous origins, including early and late ones [9, 12]. We confirmed that origin replication timing was altered in our *rpd3Δ* sample by examining origin activation of the early origin ARS305 and the late origin ARS603 during S phase as described in Additional file 5: Figure S4. Thus, 2D gel analysis indicated that when *RPD3* was deleted the bubble arc for ARS305 was most prominent at 30 min, whereas in the wild-type strain the maximum was observed at 45 min (Additional file 5: Figure S4A). In addition, we examined the position of the replication forks by monitoring the association of the Replication Protein A (RPA), a single-strand DNA-binding protein that has been shown to match the location of the replisome [32]. Chromatin immunoprecipitation (ChIP) experiments showed that the association of Rpa1 peaked at 5 kb away from ARS305 in *rpd3Δ* cells while in wild-type cells Rpa1-association was restricted to the origin position (Additional file 5: Figure S4B). Since origin efficiency seems comparable in both strains, we concluded that *ARS305* fires earlier in the mutant strain and therefore that more DNA will be synthesized before dNTP levels are depleted by HU. These data are in agreement with previous observations showing that the firing of ARS305 is advanced in *rpd3* mutants [9]. When we examined the activation of ARS603 we detected initiation structures (Additional file 5: Figure S4A), indicating that in *rpd3Δ* mutants late origins can escape checkpoint inhibition to a certain extent, as described [10]. However, the lower bubble arc signal in ARS603, suggested that late origin firing before critical dNTP depletion was taking place in a small proportion of cells. Therefore, replication timing was altered in *rpd3* mutants, and so was the nucleosomal organization around origin regions.

Then we took the opposite approach, and we analyzed the nucleosome profile of a described set of origins (Additional file 1: Table S2) with advanced replication in the absence of Rpd3 [12]. Additional file 4: Figure S3C showed similar variations in the NDR-width and occupancy of flanking nucleosomes as described in Figures 4A-D-E. These results strongly suggest that nucleosome remodelling in *rpd3* mutants is related with changes in replication timing.

Taken together, our results indicate that there is a correlation between changes in histone acetylation and replication timing with local modifications in origin architecture. Despite the fact that early and late specific chromatin patterns are conserved in *rpd3Δ* mutants, the remodelling of key origin features such as the NDR and -1 /+1 nucleosomes leads to a more open conformation. Although it has been speculated that changes in histone acetylation can influence replication timing by modifying chromatin structure [9, 10], our results demonstrate for the first time that higher levels of histone acetylation caused by the deletion of the deacetylase *RPD3* increase NDR opening.

### Origin architecture is dynamic during activation

As mentioned in the Introduction, origin activation involves the modification of chromatin-bound replicative complexes and we wondered whether this process might entail modifications in origin structure during S phase. To explore this possibility, we performed a genome-wide analysis of the dynamics of chromatin organization at replication origins before and after origin activation by comparing the nucleosomal pattern of synchronized populations in G1 and cells proceeding into S-phase in the presence of hydroxyurea (HU). The slow movement of replication forks in the presence of HU facilitates the analysis of replication dynamics and nucleosome organization during S-phase. Additionally, late origin firing is inhibited in the presence of HU by the replication checkpoint [33, 34], providing a system to compare chromatin organization in active or inactive origins within the same replication cycle.

**Figure 5:**
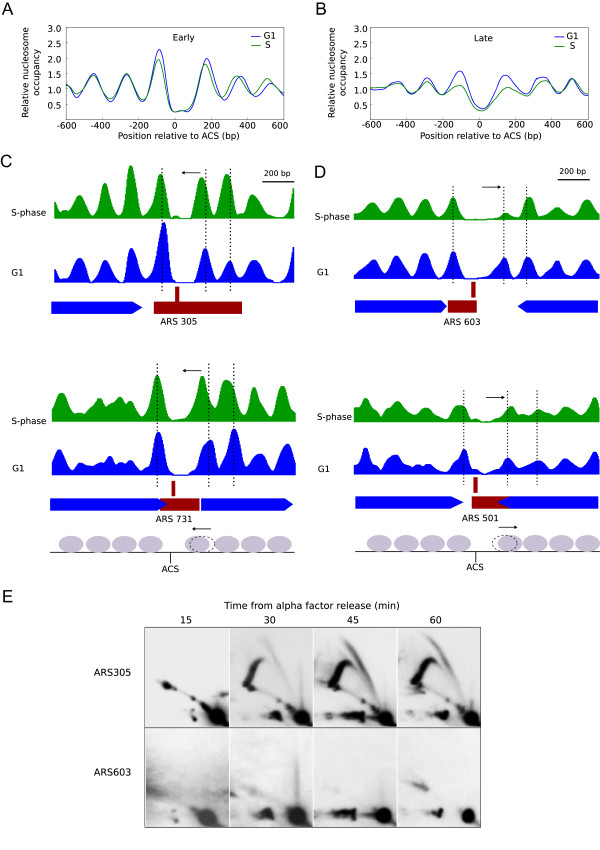
**Origin architecture is remodelled during activation.** Average nucleosomal profiles from early **(A)** and late **(B)** origin groups in G1 (blue line) and S-phase (green line). **(C)**, Nucleosome patterns of the early origins ARS 305 (top panel) and ARS 731 (bottom panel) during G1 (blue) and S-phase (green). Genes, replication origins and ACS are represented as in Figure 3. Dotted lines indicate the center of the nucleosomes flanking the origins during G1. Nucleosome remodelling at early origins is summarized in a schematic diagram where grey ovals represent nucleosomes in S-phase and the dotted nucleosome indicates the position of the +1 N (the first nucleosome downstream of the ACS) in G1. Nucleosome displacement is represented by a black arrow. **(D)**, As in C but for the late origins ARS 603 and ARS 501. **(E)**, Neutral/neutral 2D gel analysis was performed to analyze ARS305 and ARS603 activity. Genomic DNA was prepared from cells released from α-factor arrest into YPD + 0.2 M HU and collected at 15, 30, 45 and 60 min.

The analysis of nucleosome distribution in G1 and S-phase samples revealed that the majority of nucleosomes seem to occupy the same positions before and after replication initiation (see analysis of 8 Kb genomic regions at the ARS305 and ARS501 loci in Additional file 6: Figure S5). We noted that in early origin regions, and not in late ones, the average nucleosome signal was substantially higher during S phase (Additional file 6: Figure S5A). When the differential between the nucleosomal signals from the S and G1 samples was represented (grey profile) a major S-phase signal coinciding with early origin regions was corroborated (Additional file 6: Figure S5B). Higher nucleosome signals would be expected at those regions due to an increase in DNA copy number after replication from early origins. In fact, analysis of the ARS305 region indicated that a major S-G1 differential signal extended for 2–2.5 kilobases on either side of the origin (Additional file 6: Figure S5B), coinciding with the previously described length of replication intermediates accumulated at HU-blocked replication forks [33, 35]. Indeed, when nucleosome signals were corrected for copy number (see Methods), the G1 and S-phase signals in early origin positions became similar, while late origins regions remained unchanged (Additional file 6: Figure S5C). Therefore, the location and extent of a region with elevated nucleosome signals in S-phase relative to G1 mark out origin activation and newly replicated chromatin, and hence nucleosome mapping by deep sequencing is a suitable approach for analyzing both chromatin and replication dynamics at genomic scale.However, a careful comparison of the average nucleosome configuration in replication origins regions revealed that origin structure changed from G1 to S phase, and remarkably, different changes were observed in early and late origin groups (Figure 5). In early origins, +1, +2 and +3 nucleosomes were shifted to the left in S-phase cells (green line) compared to G1 cells (blue line) (Figure 5A). By contrast, in late origins, the NDR print was wider in the S-phase sample and, in this case, a clear reduction in occupancy of the -1 and +1 nucleosomes was also observed (Figure 5B).This nucleosome rearrangement was also detected in individual examples such as the early origins, ARS305 and ARS731, and late origins, ARS603 and ARS501 (Figure 5C and 5D). The high resolution achieved in our nucleosome maps was able to detect modifications at individual nucleosome positions and, in fact, early origins showed a clear displacement of the first nucleosomes opposite to the ACS towards the inner part of the NDR during S-phase (Figure 5C), while the rest were maintained at the same positions as in G1. By contrast, although changes in nucleosome positions were also restricted to the vicinity of the ACS in late origins, nucleosome displacement was directed outside the NDR and the occupancy of +1 nucleosome was drastically reduced in late origins such as ARS603 (Figure 5D).

The above observations revealed that the structure of the NDR is dynamic during replication but we then wondered why NDR-modulation would be different at early and late origins during S phase. We reasoned that the observed changes might be linked to origin activation, which in HU should take place exclusively at early origins. To confirm the unique activation of early origins in our experimental conditions, we performed 2D gel analysis of DNA replication intermediates on ARS305 and ARS603 as previously described [36]. In ARS305, the replication bubble arc indicated origin activation (Figure 5E). By contrast, when the membranes were re-probed to analyze ARS603 activation, no initiation structures at this late origin were observed. Therefore, one possibility was that the NDR-conformation would be different in origins before and after origin firing (early origin = post-firing state or late origin = pre-firing state in S phase in the presence of HU).

**Figure 6:**
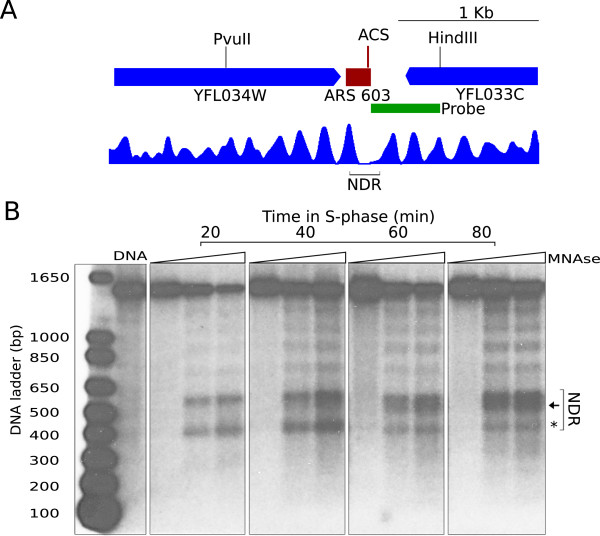
**The NDR is modified along S phase. (A)**, ARS603 is represented by a red rectangle and flanking genes, YFL034W and YFL033C, by blue ones. Vertical bars indicate sites for digestion with restriction enzymes PvuII and HindIII. Green rectangle represents the end-terminal probe. The nucleosomal profile from the G1 sample is presented below. **(B)**, Naked DNA and chromatin from wild-type cells collected at 20, 40 60 and 80 min after α-factor arrest and release into YPD were digested with increasing amounts of MNase (indicated with triangles) and subsequently digested with PvuII and HindIII. Samples were electrophoresed, transferred onto a membrane and hybridized. A ladder of polynucleosomes is observed with a prominent region of hypersensitivity to MNase corresponding to the NDR (indicated in brackets in **A**, **B**). The black arrow and asterisk point to the changes in chromatin organization observed in the NDR at later time points.

One prediction of this hypothesis is that the NDR would be modulated at replication origins at their time of origin firing. To test this possibility, we compared the nucleosome organization of the *ARS603* region before and after origin activation during an unperturbed S phase (Figure 6 and Additional file 7: Figure S6) by micrococcal nuclease (MNase) analysis. In this experiment, G1-synchronized cells were released into S phase, and different samples were taken from 20 to 80 minutes (Additional file 7: Figure S6). In each sample, chromatin was digested with increasing amounts of MNase, followed by digestion with an appropriate restriction enzyme and Southern hybridization with a terminal probe. The results shown in Figure 6 indicated that the NDR region colocalized with two MNase-hypersensitive sites flanking a protected middle region and, importantly, the lower MNase site was reduced in intensity (asterisk) while the upper MNase-hypersensitive zone was expanded (arrow) from 40 minutes onwards, coinciding with the activation time of late origins (Additional file 7: Figure S6B).

We conclude that origin nucleosomal configuration is dynamic during the cell cycle and propose that origin activation is associated with local changes in chromatin organization. Changes in chromatin architecture at origins seem to be intimately linked to their timing of activation and thus chromatin modulation in early and late origins is separated temporally. It is possible that changes in chromatin organization in origins might be required to allow the correct assembly/disassembly of replication factors before and after origin activation.

## Discussion

To elucidate the role of chromatin structure in the timing of origin firing in budding yeast, we conducted a high-resolution analysis of genome-wide nucleosomal organization at origins. Our results revealed distinctive nucleosomal configurations in early and late replication origins. The high resolution of our nucleosome maps, together with the independent analysis of early and late origins clusters were critical to disclose these different nucleosomal architectures, emphasizing the notion that the average nucleosome profile of all replication origins hides important variations in origin structure.

Previous studies have suggested that genomic regions upstream from genes tend to replicate early, while regions containing a gene end tend to replicate late [23]. In agreement with this, our analysis indicates that there is a strong coincidence of the major late patterns (5&6) at convergent IGR that contain two gene ends, and moreover, they seem to be almost completely excluded from regions that do not contain any gene end (divergent IGR). The situation seems to vary further for early origin patterns, because these can be found in regions that coincide with transcription initiation or termination, or both. However, it is unclear why late origin patterns are highly represented at convergent IGR. It is possible that a higher rate of transcription termination at convergent IGR may interfere with nucleosome stability, leading to the lower occupancy observed in late origin patterns. This possibility is supported by the fact that other convergent IGR, non-associated with replication origins, also presented low nucleosome occupancy.

Alternatively, the co-localization of origins and transcription initiation sites (TSS) may promote early firing. In this sense, replication origins from several organisms frequently contain transcription factor-binding sites [37], arguing for a role of TF in origin regulation. In *S. cerevisiae*, the transcription factor Abf1 seems to help origin activation [38], and importantly, the transcription factor Fkh is involved in early origin firing [11, 17]. Therefore, it is possible that origins located in the vicinity of a promoter would be more likely to bind Fkh1/2 transcription factors, which can strongly influence the selection of these origins for early firing. Supporting this possibility, 81% of early origins enriched with Fkh1/2-binding sites were in IGRs containing at least one promoter, while only 19% were in promoter-free regions. However, only 25.6% of the early origins analyzed contain Fkh-binding sites, suggesting that the role of TF in origin activation might not be exclusive to Fkh and that other TFs could also contribute to early firing in other origins. In fact, binding sites for other transcription factors have been found close to replication origins and have been implicated in origin activation in both yeast and mammals [39–42]. Future examination of the interactions between transcription and replication factors should provide important information about this issue.

Regarding the distribution of Fkh-binding motifs, the overlapping of predominant sites with the NDR origin feature suggests that the location of Forkhead sites within an open/accessible chromatin region is important for origin regulation. In addition, the particular localization and spacing of Fkh sites within NDR raise the possibility that the binding of TFs such as Fkh1/2 might also contribute to the maintenance of a nucleosome-free region and to the positioning of proximal nucleosomes, similar to the previously described role of ORC-binding [25].

The fact that both ORC and Fkh1/2-binding sites are restricted to the NDR suggests that the NDR is critical for accommodating functional complexes in the origins. The average NDR size from origin patterns 1–6 varies from 193 to 129 bp, indicating that different NDR widths are valid for origin activity. However, classes 1 and 2, with the broadest NDRs, and classes 5 and 6, with the narrowest NDRs, generally correlate with early and late replication respectively, indicating that NDR width might influence the timing of origin replication.

We propose that the different affinities of initiation factors for early and late origins would be determined by their different origin structure. Thus, initiation factors might have a higher affinity for early origins that generally adopt class 1 and 2 conformations and show broader and better defined NDRs. The importance of an appropriate NDR width in origin timing is highlighted by the observation that previously described dormant origins such as ARS301, ARS302, ARS303 and ARS320 show either a closed NDR or an extremely small NDR (97 bp) (Additional file 8: Figure S7). Dormant origins bind a pre-RC and are licensed in G1 [4], and if given enough time they are competent to initiate replication [43, 44]. This small NDR size probably imposes a critically low affinity for regulatory factors in these origins, being excluded for firing in a normal replication cycle. The higher occupancy by -1/+1 nucleosomes in early origins suggests that the interaction of the initiation factors with the origin region or with some components of the pre-RC complex might be facilitated or mediated by interaction with these nucleosomes. In fact, previous studies have reported an interaction between transcriptional regulators and +1 and +2 nucleosomes [45, 46].

Our results also revealed that origin architecture was actively modulated during origin firing. To visualize replication-dependent origin remodelling it is important to compare high-resolution maps from synchronized G1 and S samples, perhaps explaining why it passed unnoticed in previous studies performed on asynchronous cultures and/or with tiling microarrays [23, 24]. An interesting possibility is that origin structure might be influenced by the different replication complexes or factors bound to the origin before and after origin firing. This is in agreement with the fact that some pre-RC components and/or initiation factors are bound and released from chromatin before and after origin activation [2, 3]. Interestingly, some initiation factors such as Sld3 and Cdc45 can be detected specifically at early firing origins during G1 [11, 18, 19]. These essential initiation factors are present in the cell in limiting amounts [15, 16], and it has been suggested that they are recycled from early to late origins during S phase. Therefore, the dynamic of nucleosomes and replication proteins during the process of origin activation might be related, and we are currently examining this possibility.

In addition, we also examined the contribution of histone acetylation to early origin firing at the level of chromatin organization. Our data indicate that increased levels of histone acetylation around origins induce local modifications in chromatin organization, creating a more accessible structure. One possibility is that histone acetylation might weaken histone/DNA interactions, diminishing the chromatin association of ACS-flanking nucleosomes and altering NDR formation. The resulting open conformation in origins might facilitate, either directly or indirectly (through interaction with other chromatin-bound factors), the recruitment of critical replication factors to origins.

Taken together, the results reported argue for an important contribution of the chromatin structure to origin selection and firing, and help to understand the interplay between replication regulators such as Fkh1/2, epigenetic modifications, and chromatin structure.

## Conclusions

Coordinated origin firing is important to ensure complete replication of the entire genome and recent evidences suggest that the replication temporal program can influence genome stability [47, 48]. In this work, we have analyzed the role of chromatin structure in replication timing at the level of nucleosome organization. Our data highlight the importance of chromatin features, such as the NDR and nucleosomes +1 and -1, in the regulation of origin activation and timing. Thus, early origins present broader NDR features and a high occupancy of flanking nucleosomes while tighter NDR and a lower occupancy appear in late origins. Besides, factors involved in the establishment of origin timing such as histone deacetylation induce local modifications in these key origin features, leading to a more open origin conformation. Similarly, Fkh transcription factors seem to be in close relationship with origin structure, as suggested by the overlap of predominant Fkh-binding sites with the NDR origin feature.

Origin activation is also associated with local changes in chromatin organization. Although the specific chromatin conformations of origins are already established in G1, origin structure is dynamic during origin activation. It has been described that chromatin structure affects origin function [25], and it would be possible that origin architecture can also affect the affinity and efficiency of the formation of replication complexes and, therefore, influence the timing of origin firing.

## Methods

### Yeast strains and growth conditions

The strains used in this study are described in Additional file 9: Table S1 and derived from W303-1a (*MAT***a***ade2-1 ura3-1 his3-11 15 trp1-1 leu2-3 112 can1-100*). Yeast cultures were grown in YP medium (1% yeast extract, 2% bacto peptone) supplemented with 2% glucose (YPD) at 23°C to a final concentration of 0.7 × 10^7^ cells/ml. Cells were synchronized in G1 phase by adding 5 μg/ml α factor (αF) for one generation time and released into fresh medium in the presence/absence of 0.2 M hydroxyurea (HU) depending on the experiment.

### Preparation of mononucleosomal DNA

Previously published nucleosome maps were mostly obtained from asynchronous cultures, which would make difficult to detect nucleosome dynamics during different stages of the cell cycle. To overcome this limitation, we generated nucleosome maps from G1 and S-phase synchronized samples. Thus, 2 × 10^9^ cells were collected at G1 and at 60 minutes in S-phase in the presence of 0.2 M HU for preparation of mononucleosomal DNA and for ChIP experiments (see below). Preparation of mononucleosomal DNA was adapted from [49]. Spheroplasts were induced by treatment with 10 mg of zymolyase 20 T for 10 min at 30°C, and mononucleosomal fragments were generated by digesting DNA with 600 or 450 units/ml (WT and *rpd3* strains respectively) of micrococcal nuclease (MNAse) at 37°C during 10 minutes. The amount of MNase was optimized experimentally for each strain to generate an 80:20 ratio of mononucleosomes to dinucleosomes, as described [50].

### Sequencing and nucleosome maps representation

Mononucleosomal DNA was sequenced using the single-end sequencing protocol in an Illumina Genome Analyzer IIx. All sequencing data are deposited in the Gene Expression Omnibus (GEO) database under the accession number [GSE54377]. 23117530 to 28511688 single reads 40 nucleotides long were aligned with the reference genome of S. cerevisiae S288C (R64-1-1 assembly 3/2/2011). For the alignment we used Bowtie with no mismatches allowed. and the average genome coverage obtained was from 76 to 95-fold. This high level of resolution is a critical factor to detect subtle local modifications at individual nucleosome positions. The sequencing coverage for every nucleotide was normalized by the average genomic coverage. The nucleosome positioning profile was generated as described [51]. Briefly, the average spacing between boundary peaks for individual nucleosomes was calculated from the smoothed signal generated with Python “Pywavelets” (with the multilevel 1-D biorthogonal wavelet decomposition/reconstruction tool). Then, the midpoint position of each nucleosome was defined and the resulting combined profile was wavelet-smoothed. For the copy number correction shown in Additional file 6: Figure S5, the ratio of mapped reads from the G1 and S-phase nucleosomal samples to the naked-DNA samples from the same time points (SRR398641 and SRR398652 files deposited in SRA database) was calculated. The number of reads from the G1 and S naked-DNA samples were corrected to obtain an average number value across the genome of one, as previously described in replication timing profiles analysis [52]. The resulting signal was then smoothed with a 1000 bp window, step 1 bp.

### Clustering, analysis of NDRs, TSS and search for Fkh motif

A list of replication origins with annotated ACS in the +/-20 nt limits of each origin was obtained from OriDB and those origins that mapped on a repetitive region were excluded. To generate the average nucleosome profiles, the nucleosome maps were aligned relative to the ACS and were oriented with respect to the T-rich strand. The nucleosome profiles of the [-600, 600] nt, centered on each ACS sequence, from the WT G1 sample were clustered using a k-mean clustering function with 10.000 iterations. We used 4 to 8 groups in our analysis, and six groups were finally chosen because they accomplished the best consensus between cluster size and information. Nucleosome-depleted regions (NDRs) were defined as regions spanning at least 80 nucleotides as described [53], with a normalized sequence coverage lower than 0.4. The annotations of [54] were used as a TSS reference. The whole genome was searched for the FKH motif (RTAAAYA) previously described [17] by using the tool FIMO with a maximum threshold of 0.001. We cured it by deleting all non-strict coincidences with the motif.

### Two-dimensional gel analysis

DNA was isolated from 2 × 10^9^ cells using a G-20 column (QIAGEN) according to the manufacturer’s instructions. DNA fragments digested using XbaI and EcoRI (Fermentas) or BamHI and NcoI (Fermentas) were separated by neutral/neutral two-dimensional agarose gel electrophoresis as described [55].

### Chromatin Immunoprecipitation (ChIP) and quantitative real-time PCR (qPCR)

ChIP extracts were prepared from 7.5 × 10^8^ cells as described [56]. Immunoprecipitation was carried out overnight with 5 mg of anti-PK antibody (SV5-Pk1 Fisher Scientific Pierce) coupled to magnetic beads (Dynabeads, Invitrogen). Uncoupled Dynabeads were used for background controls. The DNA fragments recovered from the whole-cell extract and ChIP samples were quantified by real-time PCR using the BioRad-CFX96 system. The q-PCR signals obtained from the ChIP samples were normalized by the signals obtained from an input sample (percent IP). Primer sequences and amplification reactions are indicated in Additional file 9: Table S1. Primers were tested to achieve amplification curves with a slope of 3.3 ± 10% and R2 value >0.99. All real-time PCRs were performed in duplicate with SYBR Premix Ex Taq (TaKaRa) in two independent experiments.

### Digestion with MNase and indirect end-labelling analyses

2×10^9^ cells were permeabilized as described above and spheroplasts were split into six fractions in NP-buffer and treated with increasing amounts of MNase (Fermentas) (0, 0.5, 1, 1.5, 2 and 3 units/ml) for 10 minutes at 37°C. DNA was purified and quantified. For each sample, 2.5 μg of DNA were digested with HindIII and PvuII enzymes (Fermentas), separated by electrophoresis in a 1.5% agarose gel, blotted and hybridized to an specific end-terminal probe.

### Availability of supporting data

The data sets supporting the results of this article are available in the [GSE54377] repository, [http://www.ncbi.nlm.nih.gov/geo/query/acc.cgi?acc=GSE54377].

## Electronic supplementary material

Additional file 1: Table S2: Origin data set. (XLS 106 KB)

Additional file 2: Figure S1: Early and late replication origins are preferentially located in different IGR. **(A)**, Average nucleosome profiles of early (blue line) and late (green line) replication origins for each of the six groups established by the k-means clustering shown in Figure 1A. **(B)**, Frequencies of expected (grey) and observed early (blue) and late (green) replication origins in IGR between tandem, divergent and convergent transcripts. Frequencies of IGR between tandem, divergent and convergent transcripts are 50%, 25% and 25% respectively. **(C)**, Average nucleosomal profiles from NDR in tandem, divergent and convergent IGR that are non associated with replication origins. (PDF 109 KB)

Additional file 3: Figure S2: The nucleosomal landscape in *S. cerevisiae* is largely maintained in the absence of Rpd3. **(A)**, Aggregated nucleosomal profiles of WT and *rpd3Δ* mutants from G1 (blue and red lines, respectively) and S-phase cells (green and yellow lines) were aligned to the midpoint position of the +1 nucleosome (+1 N), as described in Soriano et al. [51], and to the transcription start site (TSS). The coordinates of TSS have been reported by Lee et al. [54]. **(B)**, Nucleosome patterns across a chromosome VII region from WT (blue) and *rpd3Δ* cells (red) as in Figure 3. (PDF 100 KB)

Additional file 4: Figure S3: Analysis of nucleosomal profiles in early and late replication origins in the absence of Rpd3. **(A)**, As in Figure 1C but for *rpd3Δ* cells. **(B)**, Comparative nucleosomal profiles from WT and *rpd3Δ* cells in G1 (left panel) and S-phase (right panel). **(C)**, Aggregated nucleosomal profiles of 51 Rpd3-regulated origins described in Knott et al., [12] from WT (blue) and *rpd3Δ* (red) cells were aligned relative to the ACS. **(D)**, **(E)**, Nucleosome patterns around the origins ARS 1410 and ARS 1413 from WT (blue) and *rpd3Δ* samples (red). Black arrows point to nucleosomes affected in the absence of Rpd3. (PDF 154 KB)

Additional file 5: Figure S4: Replication dynamics in HU-treated *rpd3Δ* cells. **(A)**, Analysis of origin activation by two-dimensional gel electrophoresis of ARS305 and ARS603 origins in wild-type and *rpd3Δ* cells. Genomic DNA was prepared from cells released from α-factor arrest into YPD + 0.2 M HU and collected at 15, 30, 45 and 60 min. Black arrows indicate the sample with maximum intensity of the bubble arc in ARS305 (top panels). Arrows point to bubble arcs in ARS603 (bottom panels). **(B)** ChIP analysis of Rfa1-PK was performed in wild-type (left graph) and *rpd3Δ* cells (right graph). Cells with PK-tagged Rfa1 were synchronized in G1 and released into rich medium containing 0.2 M HU for 60 min. ChIP was performed with a-PK antibody. Histograms represent the percentage of immunoprecipitated DNA relative to the input. The PCR primers pairs correspond to the ACS and adjacent regions at the early origin ARS 305. Standard deviation bars are indicated. (PDF 3 MB)

Additional file 6: Figure S5: Higher nucleosome signals correlate with origin activation. **(A)**, Average nucleosomal profiles from early and late origin groups in G1 (blue line) and S-phase (green line). **(B)**, Nucleosome patterns across 8 kilobases of the *S. cerevisiae* genome encompassing the early origin ARS 305 (top) and the late origin ARS501 (bottom) from G1 (blue) and S-phase (green) cells. The differential signal between S-phase and G1 data is shown in grey. Genes, replication origins and ACS are represented as in Figure 3. **(C)**, As in A, but nucleosome signals were corrected for copy number as described in Methods. (PDF 130 KB)

Additional file 7: Figure S6: Nucleosome and replication analysis during an unperturbed S-phase. Wild-type cells with PK-tagged Rfa1 were synchronized in G1 and released into rich medium. Samples were collected at the indicated time points for flow cytometric, ChIP and MNase analysis. **(A)**, DNA content was measured by flow cytometry **(B)**, Immunoprecipitated DNA was analyzed for the presence of ARS305 (circles), ARS609 (squares) and ARS501 (triangles) sequences by qPCR as described above. **(C)**, Complete gel corresponding to Figure 6B, where only the three first lanes were shown for each time point. (PDF 782 KB)

Additional file 8: Figure S7: Dormant origins present closed or small NDR. Nucleosome patterns across a chromosome III region including ARS301, ARS302, ARS303 and ARS320. Genes, replication origins and ACS are represented as in Figure 3. (PDF 39 KB)

Additional file 9: Table S1: Yeast strains and primer sequences. (XLS 18 KB)
