# Supplementary figures and images for: Different nucleosomal architectures at early and late replicating origins in Saccharomyces cerevisiae
Source: BMC Genomics. 2014 Sep 13;15(1):791. doi: 10.1186/1471-2164-15-791 (PMC4176565; doi:10.1186/1471-2164-15-791)

A

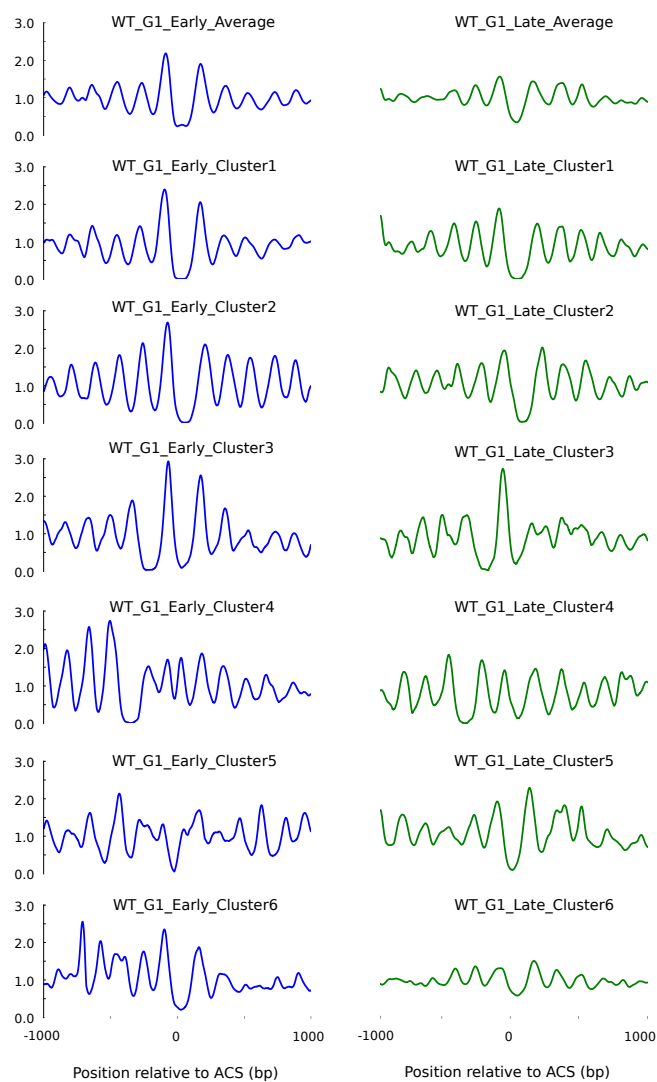

B

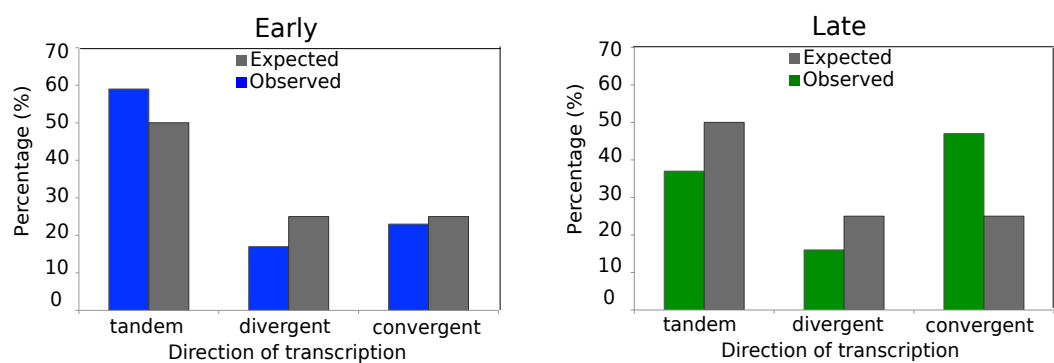

C

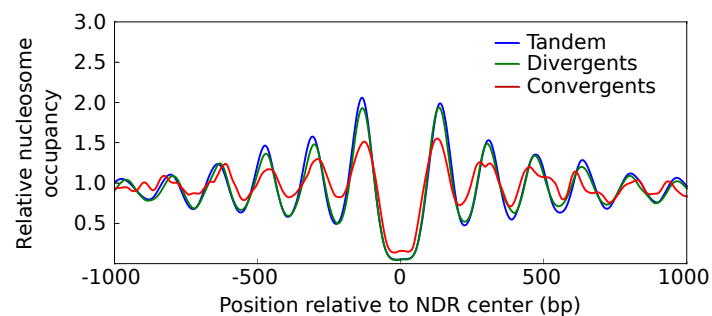

Supplementary Figure 1

Supplement: Supplementary file 2 — Additional file 2: Figure S1: Early and late replication origins are preferentially located in different IGR. (A), Average nucleosome profiles of early (blue line) and late (green line) replication origins for each of the six groups established by the k-means clustering shown in Figure 1A. (B), Frequencies of expected (grey) and observed early (blue) and late (green) replication origins in IGR between tandem, divergent and convergent transcripts. Frequencies of IGR between tandem, divergent and convergent transcripts are 50%, 25% and 25% respectively. (C), Average nucleosomal profiles from NDR in tandem, divergent and convergent IGR that are non associated with replication origins. (PDF 109 KB) [file 12864_2014_6476_MOESM2_ESM.pdf]

A

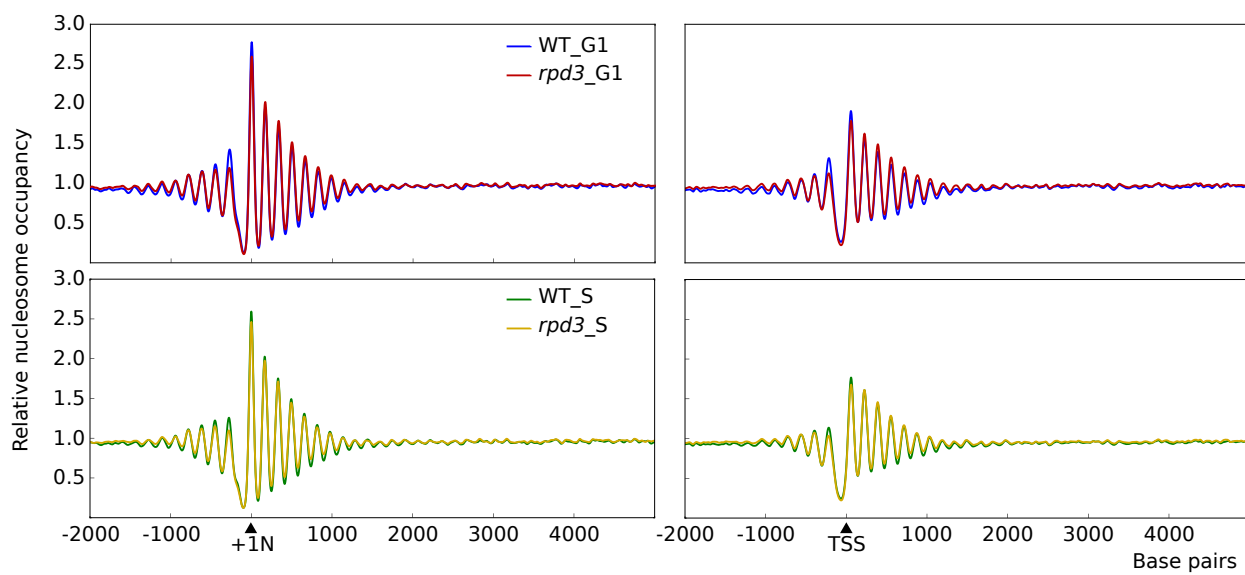

B

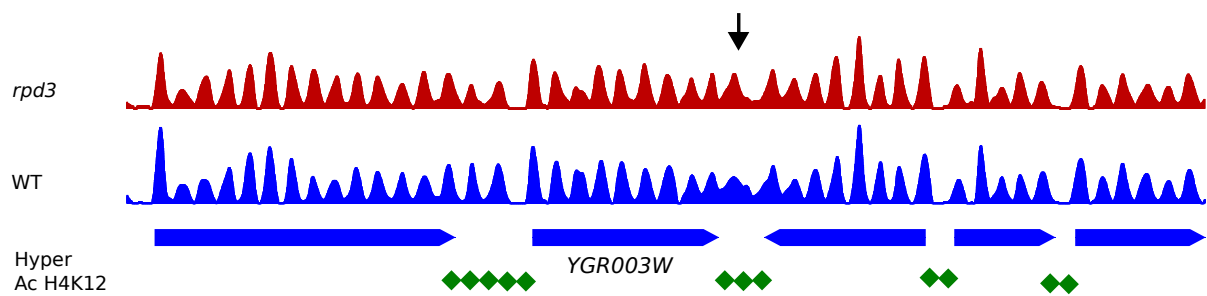

Supplementary Figure 2

Supplement: Supplementary file 3 — Additional file 3: Figure S2: The nucleosomal landscape in S. cerevisiae is largely maintained in the absence of Rpd3. (A), Aggregated nucleosomal profiles of WT and rpd3Δ mutants from G1 (blue and red lines, respectively) and S-phase cells (green and yellow lines) were aligned to the midpoint position of the +1 nucleosome (+1 N), as described in Soriano et al. [51], and to the transcription start site (TSS). The coordinates of TSS have been reported by Lee et al. [54]. (B), Nucleosome patterns across a chromosome VII region from WT (blue) and rpd3Δ cells (red) as in Figure 3. (PDF 100 KB) [file 12864_2014_6476_MOESM3_ESM.pdf]

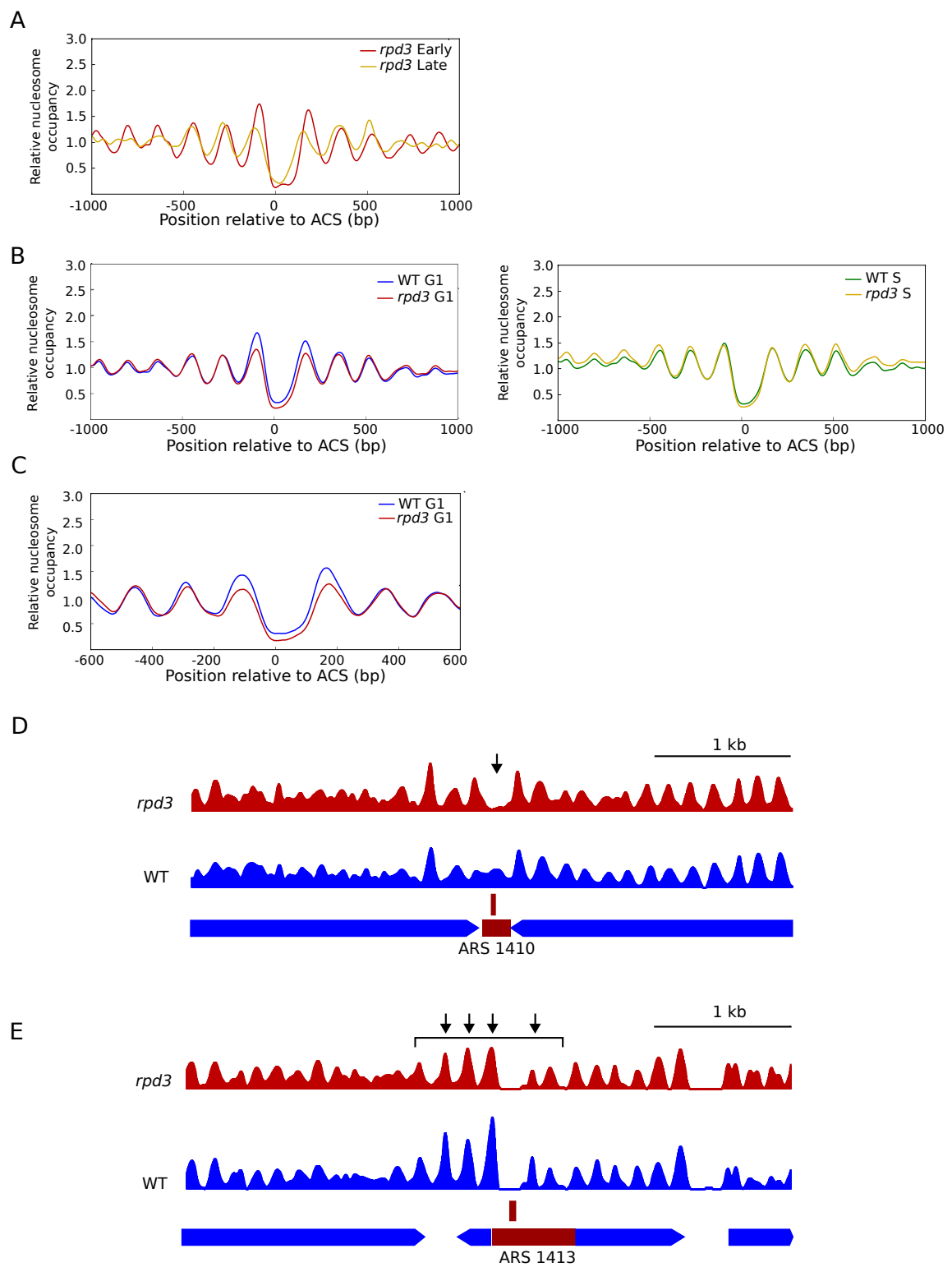

Supplementary Figure 3

Supplement: Supplementary file 4 — Additional file 4: Figure S3: Analysis of nucleosomal profiles in early and late replication origins in the absence of Rpd3. (A), As in Figure 1C but for rpd3Δ cells. (B), Comparative nucleosomal profiles from WT and rpd3Δ cells in G1 (left panel) and S-phase (right panel). (C), Aggregated nucleosomal profiles of 51 Rpd3-regulated origins described in Knott et al., [12] from WT (blue) and rpd3Δ (red) cells were aligned relative to the ACS. (D), (E), Nucleosome patterns around the origins ARS 1410 and ARS 1413 from WT (blue) and rpd3Δ samples (red). Black arrows point to nucleosomes affected in the absence of Rpd3. (PDF 154 KB) [file 12864_2014_6476_MOESM4_ESM.pdf]

A

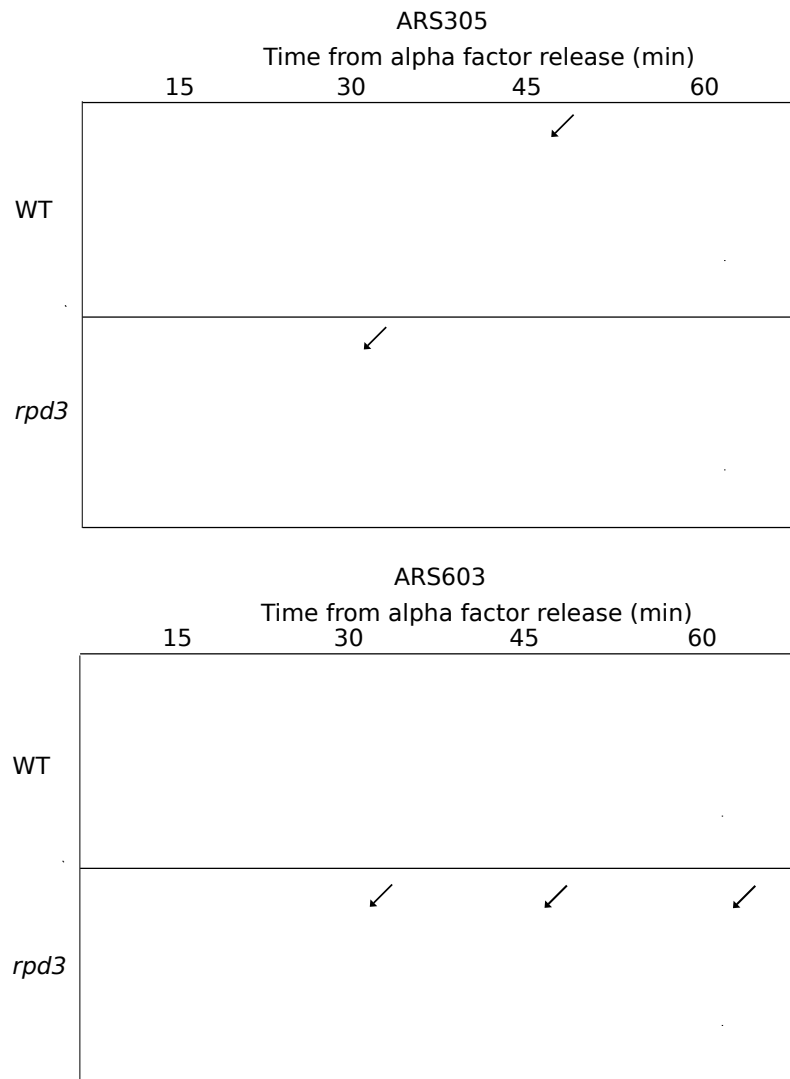

B

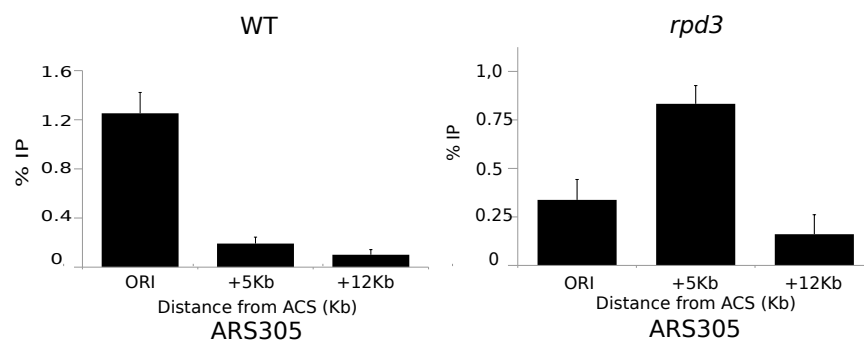

Supplementary Figure 4

Supplement: Supplementary file 5 — Additional file 5: Figure S4: Replication dynamics in HU-treated rpd3Δ cells. (A), Analysis of origin activation by two-dimensional gel electrophoresis of ARS305 and ARS603 origins in wild-type and rpd3Δ cells. Genomic DNA was prepared from cells released from α-factor arrest into YPD + 0.2 M HU and collected at 15, 30, 45 and 60 min. Black arrows indicate the sample with maximum intensity of the bubble arc in ARS305 (top panels). Arrows point to bubble arcs in ARS603 (bottom panels). (B) ChIP analysis of Rfa1-PK was performed in wild-type (left graph) and rpd3Δ cells (right graph). Cells with PK-tagged Rfa1 were synchronized in G1 and released into rich medium containing 0.2 M HU for 60 min. ChIP was performed with a-PK antibody. Histograms represent the percentage of immunoprecipitated DNA relative to the input. The PCR primers pairs correspond to the ACS and adjacent regions at the early origin ARS 305. Standard deviation bars are indicated. (PDF 3 MB) [file 12864_2014_6476_MOESM5_ESM.pdf]

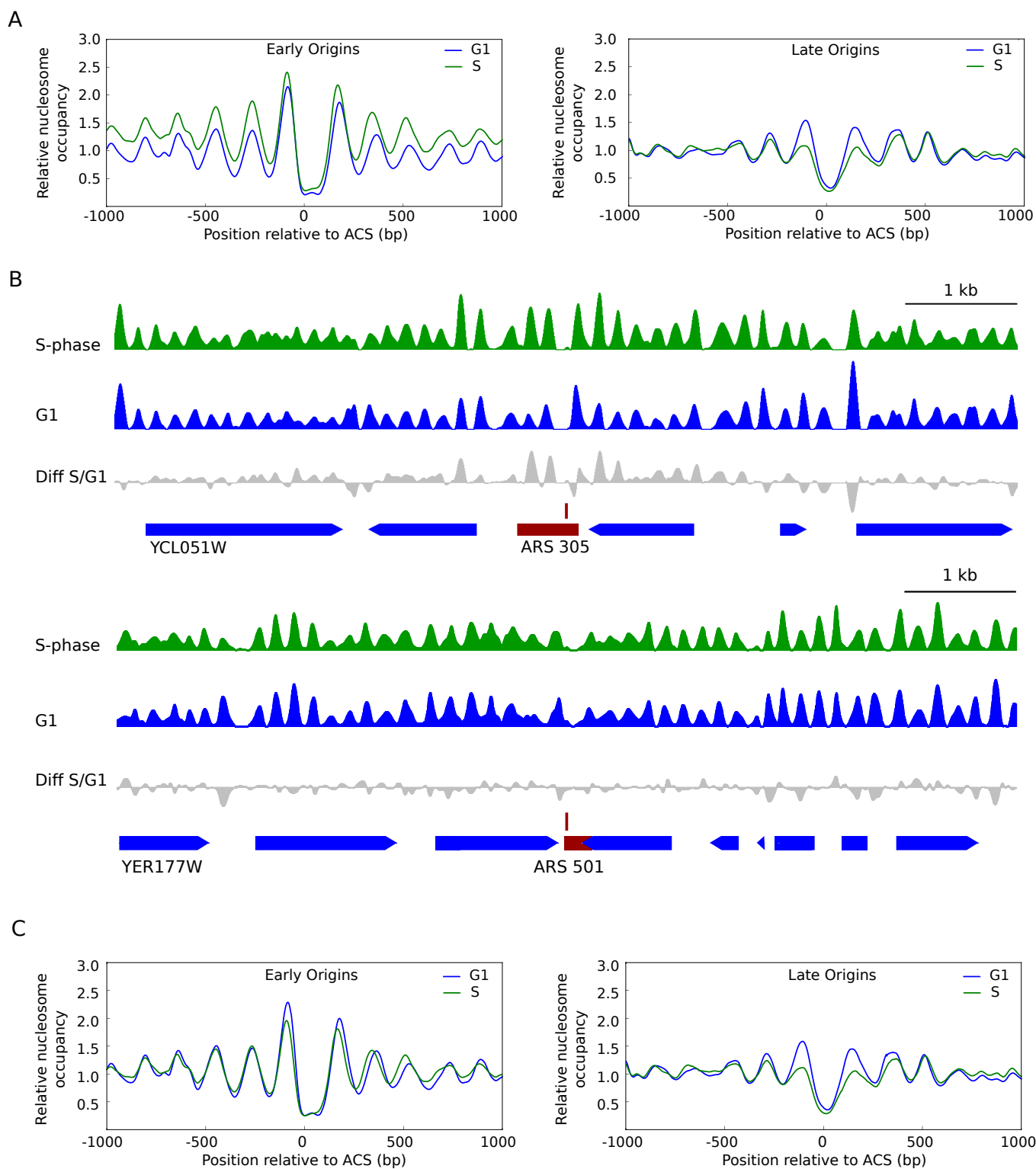

Supplementary Figure 5

Supplement: Supplementary file 6 — Additional file 6: Figure S5: Higher nucleosome signals correlate with origin activation. (A), Average nucleosomal profiles from early and late origin groups in G1 (blue line) and S-phase (green line). (B), Nucleosome patterns across 8 kilobases of the S. cerevisiae genome encompassing the early origin ARS 305 (top) and the late origin ARS501 (bottom) from G1 (blue) and S-phase (green) cells. The differential signal between S-phase and G1 data is shown in grey. Genes, replication origins and ACS are represented as in Figure 3. (C), As in A, but nucleosome signals were corrected for copy number as described in Methods. (PDF 130 KB) [file 12864_2014_6476_MOESM6_ESM.pdf]

A

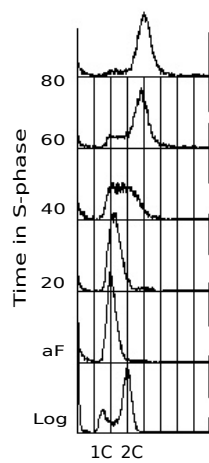

B

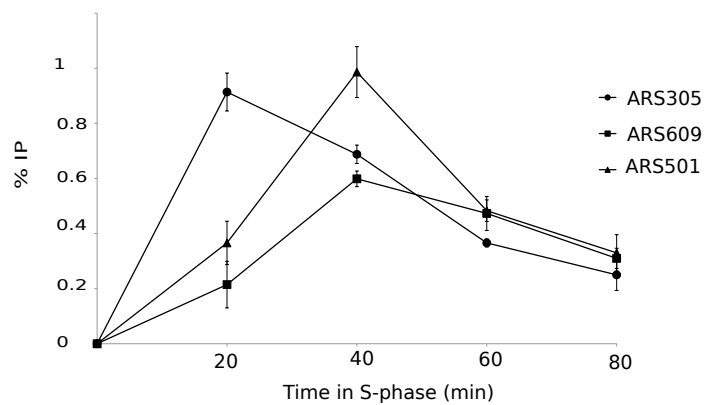

C

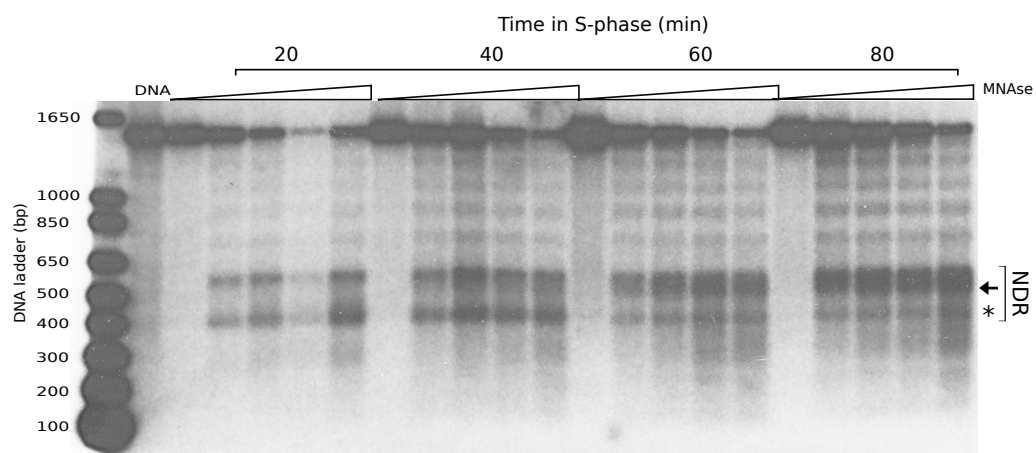

Supplementary Figure 6

Supplement: Supplementary file 7 — Additional file 7: Figure S6: Nucleosome and replication analysis during an unperturbed S-phase. Wild-type cells with PK-tagged Rfa1 were synchronized in G1 and released into rich medium. Samples were collected at the indicated time points for flow cytometric, ChIP and MNase analysis. (A), DNA content was measured by flow cytometry (B), Immunoprecipitated DNA was analyzed for the presence of ARS305 (circles), ARS609 (squares) and ARS501 (triangles) sequences by qPCR as described above. (C), Complete gel corresponding to Figure 6B, where only the three first lanes were shown for each time point. (PDF 782 KB) [file 12864_2014_6476_MOESM7_ESM.pdf]

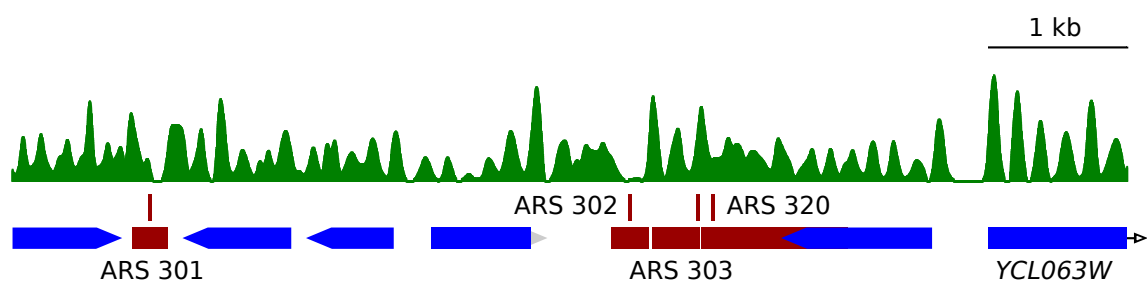

Supplementary Figure 7

Supplement: Supplementary file 8 — Additional file 8: Figure S7: Dormant origins present closed or small NDR. Nucleosome patterns across a chromosome III region including ARS301, ARS302, ARS303 and ARS320. Genes, replication origins and ACS are represented as in Figure 3. (PDF 39 KB) [file 12864_2014_6476_MOESM8_ESM.pdf]
